# B cells inhibit IL-1 family cytokine production and *Mycobacterium tuberculosis* growth in human CD14^+^ cells

**DOI:** 10.1093/immhor/vlaf046

**Published:** 2025-10-09

**Authors:** Abhinav Vankayalapati, Bharath Somasundram, Padmaja Paidipally, Karan P Singh, Ramakrishna Vankayalapati, Rajesh Kumar Radhakrishnan

**Affiliations:** Center for Biomedical Research, The University of Texas Health Science Center at Tyler, Tyler, TX, United States; Division of Infectious Diseases, Allergy and Immunology, Saint Louis University, Saint Louis, MO, United States; Center for Biomedical Research, The University of Texas Health Science Center at Tyler, Tyler, TX, United States; Department of Epidemiology and Biostatistics, School of Medicine, The University of Texas Health Science Center at Tyler, Tyler, TX, United States; Center for Biomedical Research, The University of Texas Health Science Center at Tyler, Tyler, TX, United States; Center for Biomedical Research, The University of Texas Health Science Center at Tyler, Tyler, TX, United States

**Keywords:** B cells, IL-1 superfamily, macrophages, monocytes, *Mtb*

## Abstract

The IL-1 family of cytokines produced by antigen-presenting cells plays important roles in various diseases and infections, including *Mycobacterium tuberculosis* (*Mtb*) infection. In the present study, we infected human monocyte-derived macrophages (MDMs) with *Mtb*. Then, we measured the production of IL-1 superfamily (ILSF) cytokines (8 soluble factors) and determined the effects of ILSF cytokines on *Mtb* growth via the use of recombinant cytokines and antibodies. *Mtb* infection significantly increased the production of IL-1α, IL-1β, IL-18, and IL-37 and reduced the production of IL-1Ra by MDMs. Human recombinant IL-1α, IL-1β, and IL-18 reduced *Mtb* growth in MDMs. In contrast, human recombinant IL-1Ra enhanced *Mtb* growth in MDMs. Neutralizing antibodies against IL-1α, IL-1β, and IL-18 enhanced *Mtb* growth, and neutralizing antibodies against IL-1Ra and IL-33R reduced *Mtb* growth in MDMs. B cells are known to regulate inflammation in tuberculosis (TB) granulomas. We also determined the effects of B and NK cells on ILSF cytokine production by human monocytes. Furthermore, we determined the effect of B cells on *Mtb* growth in human monocytes. B cells significantly reduced IL-1α, IL-1β, IL-6, and TNF-α production; enhanced IL-1Ra, IL-18, and IL-10 production; and inhibited *Mtb* growth in human CD14^+^ monocytes. These findings may be relevant in human TB granulomas, where B cells may regulate the balance of proinflammatory and anti-inflammatory ILSF cytokines and inhibit TB growth.

## Introduction

Tuberculosis (TB) is the second leading cause of death from a single infectious agent, killing approximately 1.3 million people annually.^1^ Alveolar macrophages are involved in the initial innate defense against various lung pathogens, including *Mycobacterium tuberculosis* (*Mtb*).^2^^,^^3^ Various cytokines released by macrophages play important roles during *Mtb* infection.^4^ In-depth analysis of cytokine release from alveolar macrophages will provide a better understanding of immune responses to *Mtb.*^5^^,^^6^

The IL-1 superfamily (ILSF) of cytokines produced by antigen-presenting cells (APCs) plays an important role in various diseases and infections, including *Mtb* infection.^7^^,^^8^ IL-1α, IL-1β, IL-18, IL-33, IL-36α, IL-36β, IL-36γ, IL-37, IL-38, IL-1Ra, and IL-36Ra are the 11 soluble factors of the ILSF of cytokines.^8^ Acute respiratory distress syndrome of the lungs, influenza, and COVID-19 are examples of diseases that involve both cytokine storms and IL-1α and IL-1β.^9^ Some viral infections of the lungs present with increased amounts of IL-33.^9^ IL-1α and IL-1β have important roles in controlling bacterial infections in mice.^10^ The ILSF of cytokines plays a major role in the immune response to TB.^11–15^ Reports have shown the utility of circulating IL-1Ra as a marker of TB.^4^ In patients with active TB, elevated levels of IL-1Ra and reduced levels of IL-1α and IL-1β after BCG stimulation have been noted.^4^ The cytokines IL-1α and IL-33 also act as damage-associated molecular patterns,^16^ and necrotic cells release IL-1α to function as an alarm for the initiation and recruitment of cytokine/chemokine and effector immune cells.^9^ IL-18 produced by alveolar macrophages and PBMCs enhances IFN-γ production and provides protective immunity against TB.^17^^,^^18^ However, the role of ILSF cytokines in *Mtb* growth in human macrophages has not been comprehensively elucidated.

T cells are known to regulate ILSF cytokine production and *Mtb* growth in APCs.^19^^,^^20^ B cells play an important role during *Mtb* infection.^21–23^ During chronic TB infection, B cells play a proinflammatory role by increasing the number of CD4^+^ T cells available so that they can generate more IFN-γ to allow for the development of granulomas.^21^ B cells can regulate the balance of pro- and anti-inflammatory responses in TB granulomas.^24^^,^^25^ Antigen-specific B cells control *Mtb* infection through the localization of T follicular helper–like cells in granuloma-associated lymphoid follicles.^26^ There is limited information available on the role of B cells in *Mtb* growth and ILSF cytokine production by human APCs.^23^^,^^27^^,^^28^

In the present study, we evaluated the production of ILSF cytokines by human monocyte-derived macrophages (MDMs) and investigated the comprehensive role of various ILSF cytokines in *Mtb* growth. We also determined the effects of B cells on ILSF cytokine production and *Mtb* growth in human monocytes.

## Materials and methods

### Ethical approval and blood sample collection

Healthy human volunteer blood samples were collected for this study, and written consent was obtained from the study participants. All studies were approved by the Institutional Review Board of the University of Texas Health Science Center at Tyler (HSC-1022) and Saint Louis University (34724 and 33195).

### Antibodies and reagents

The following antibodies and human recombinant proteins were obtained from R&D Systems: anti-ST2/IL-33R antibody (catalog # AF-523), anti-IL-1β/IL-1F2 antibody (catalog # MAB601), anti-IL-1α/IL-1F1 antibody (catalog # MAB200), anti-IL-1Ra/IL-1F3 antibody (catalog # MAB280), anti-IL-18Rα/IL-1R5 antibody (catalog # MAB840), anti-IL-36/IL-1F3 antibody (catalog # AF1078), anti-IL-37/IL-1F7 antibody (catalog # AF1975), anti-IL-38/IL-1F10 antibody (catalog # AF2427), anti-rIL-37/IL-1F7 protein (catalog # 1975-IL), mouse monoclonal IgG1 antibody (catalog # MAB002), mouse monoclonal IgG2A antibody (catalog # MAB003), and goat polyclonal IgG antibody (catalog # AB-108-C). rIL-1α (catalog # 570002), rIL-1β (catalog # 579402), rIL-18 (catalog # 592102), rIL-33 (catalog # 581802), rIL-1Ra/IL-1RN (catalog # 553902), and rIL-36Ra/IL-1F5 (catalog # 760904) were obtained from BioLegend, and the rIL-38/IL-1F10 protein (catalog # NBP2-52047) was obtained from Novus Biologicals.

### Isolation of monocytes, B cells, and NK cells

Peripheral blood mononuclear cells (PBMCs) were isolated via Ficoll–Paque gradient differential centrifugation as previously described.^29^ On day 0, CD14^+^ monocytes were isolated via magnetic beads, and their purity was checked according to the manufacturer’s instructions (Miltenyi Biotec, catalog # 130-050-201). Isolated CD14^+^ monocytes (>95% pure cells) were plated in multiple wells of a 12-well plate at 1.2–2 × 10^6^ cells/well in complete cell culture RPMI medium supplemented with 10% heat-inactivated human serum, 1% sodium pyruvate, and 1% penicillin-streptomycin. Monocytes were incubated at 37 °C in a humidified CO_2_ incubator for 5 days to allow them to differentiate into MDMs. Adhered macrophages from the same donors were washed with 1× HBSS. Some cells were used as control and others were infected with *Mtb.* Some of the *Mtb*-infected cells were treated with either recombinant proteins or neutralizing antibodies.

For some experiments, CD14^+^ monocytes were either cocultured with autologous NK cells or B cells to determine the ILSF cytokine production, *Mtb* growth, and the cell death mechanisms. We used NK cells (10 monocytes with one NK cell) as a positive control^30^^,^^31^ and 2 different concentrations of B cells (10 monocytes with one B cell or 5 B cells). PBMCs were isolated from blood, and CD14^+^ monocytes, Pan B cells, and total NK cells were separated via magnetic beads from autologous PBMCs according to the manufacturer’s instructions (Miltenyi Biotec; Pan B Cell Isolation Kit, catalog # 130-101-638 and NK Cell Isolation Kit, catalog # 130-092-657) and cocultured on the same day of the experiment (day 0). Purity of NK cells (CD3^−^NK1.1^+^; >95%) and B cells (CD3^−^CD19^+^; >92%) were checked by flow cytometry (Thermo Attune NxT and BD LSR Fortessa X-20) and used for the subsequent experiments (Fig. S1A, B).

### Macrophage/monocyte infection, coculture with B cells and NK cells, and ILSF proteins/cytokines

Some of the MDMs were infected with *Mtb* H37Rv at a multiplicity of infection of 1:2.5 in antibiotic-free medium and incubated for 2 hours at 37 °C in a humidified CO_2_ incubator. After incubation, the cells were washed with 1× HBSS for a total of 3 washes to remove extracellular bacilli and incubated with antibiotic-free RPMI medium containing 10% heat-inactivated human serum and 1% sodium pyruvate. In some experiments, recombinant proteins were added to MDMs cultured with *Mtb* at concentrations of 10 ng/mL (rIL-1α, rIL-1β, rIL-33, rIL-36Ra, and rIL-38), 100 ng/mL (rIL-1Ra and rIL-18), and 3 µg/mL (rIL-37). Concentrations for recombinant proteins were selected based on previous published studies and manufacturers recommendations.^32^^,^^33^ For neutralization experiments, concentrations were determined as per the manufacturer’s instructions and previously published studies. Neutralizing anti-IL-1α (anti-mouse IgG2k), anti-IL-1β (anti-mouse IgG1k), anti-IL-1Ra (anti-mouse IgG2k), anti-IL-18Rα (anti-mouse IgG1k), and anti-IL-33R (anti-goat IgG) antibodies or their IgG isotype controls were used at a concentration of 10 µg/mL.^34–37^

For B cell and NK cell coculture experiments, freshly isolated monocytes, B cells, and NK cells were counted via the trypan blue exclusion method. In some experiments, freshly isolated B cells were added to CD14^+^ monocytes at a concentration of 1 or 5 cells per 10 monocytes (1 × 10^6^ CD14^+^ to 1 or 5 × 10^5^ CD19^+^ B cells), and NK cells were added to monocytes at a concentration of 1 cell per 10 monocytes (1 × 10^6^ CD14^+^ to 1 × 10^5^ NK cells) on the same day that CD14^+^ cells were isolated. The cocultured cells (CD14: B or CD14: NK) were infected with *Mtb* H37Rv immediately. At 24, 48, 72, or 120 hours postinfection, the cell pellets were used to determine the bacterial burden and RNA isolation, and the cell culture supernatants were collected for various cytokine/chemokine analyses.

### Determination of the bacterial burden in *Mtb*-infected MDMs or monocytes

At 120 hours postinfection, the MDMs or monocytes were lysed in 1 mL of 7H9 broth containing 0.25% SDS and 1% BSA, serially diluted, and plated on 7H10 agar plates (Thermo Scientific, catalog # 01600) in triplicate. Colony-forming units (CFUs) were determined 21 days after incubation by counting the number of colonies on the agar plates.

### Measurement of cytokine production by ELISA

The following cytokines were measured in the cell culture supernatants: IL-6 (catalog # 430504, BioLegend), IL-10 (catalog # 430604, BioLegend), TNF-α (catalog # 430204, BioLegend), IL-1β (catalog # 437004, BioLegend), IL-37 (catalog # DY1975, R&D Systems), and IL-38 (catalog # DY9110, R&D Systems). In some experiments, IL-1 family cytokines were measured via a multiplex ELISA kit via magnetic Luminex assays (catalog # CUSTOM-LXSA-H-16 lot # C0008953, R&D Systems).

### RNA isolation and qRT-PCR

Total RNA was extracted from *Mtb*-infected MDMs or monocytes via TRIzol (Invitrogen) according to the manufacturer’s instructions. The isolated RNA was quantified and reverse transcribed (iScript Reverse Transcription SuperMix kit; Bio-Rad), and real-time PCR was performed on a Bio-Rad CFX384 instrument with iTaq Universal SYBR Green Supermix (Bio-Rad). mRNA expression was quantified via the 2^−ΔΔCt^ method, and gene expression levels were normalized to those of β-actin internal controls. The forward and reverse primers used for this study are listed in Table S1.

### Annexin V/propidium iodide staining

Isolated CD14^+^ monocytes were cocultured with B cells and stimulated with γ-*Mtb* (gamma-irradiated *Mtb* H37Rv) at 10 µg/mL. After 48 hours of incubation, the cells were collected and stained with Annexin V and propidium iodide (PI), and staining results were acquired via flow cytometry (Thermo Attune NxT and BD LSR Fortessa X-20).

### Statistical analysis

Statistical analysis was performed via Prism 10.2.00 software (GraphPad). The same donor samples were cultured in multiple wells. For statistical analysis, we have used either 2-tailed paired *t*-test or repeated-measures one-way ANOVA since the comparisons were made between the same donor samples (control vs treated) and considered as same cohort. To reduce type 1 error (false-positive), we have used post hoc Fisher least significant difference (LSD) test or Tukey multiple comparisons test. The results are shown as the mean ± SD. *P* < 0.05 was considered statistically significant.

## Results

### ILSF cytokine production by *Mtb* H37Rv–infected human MDMs

**Figure 1.**
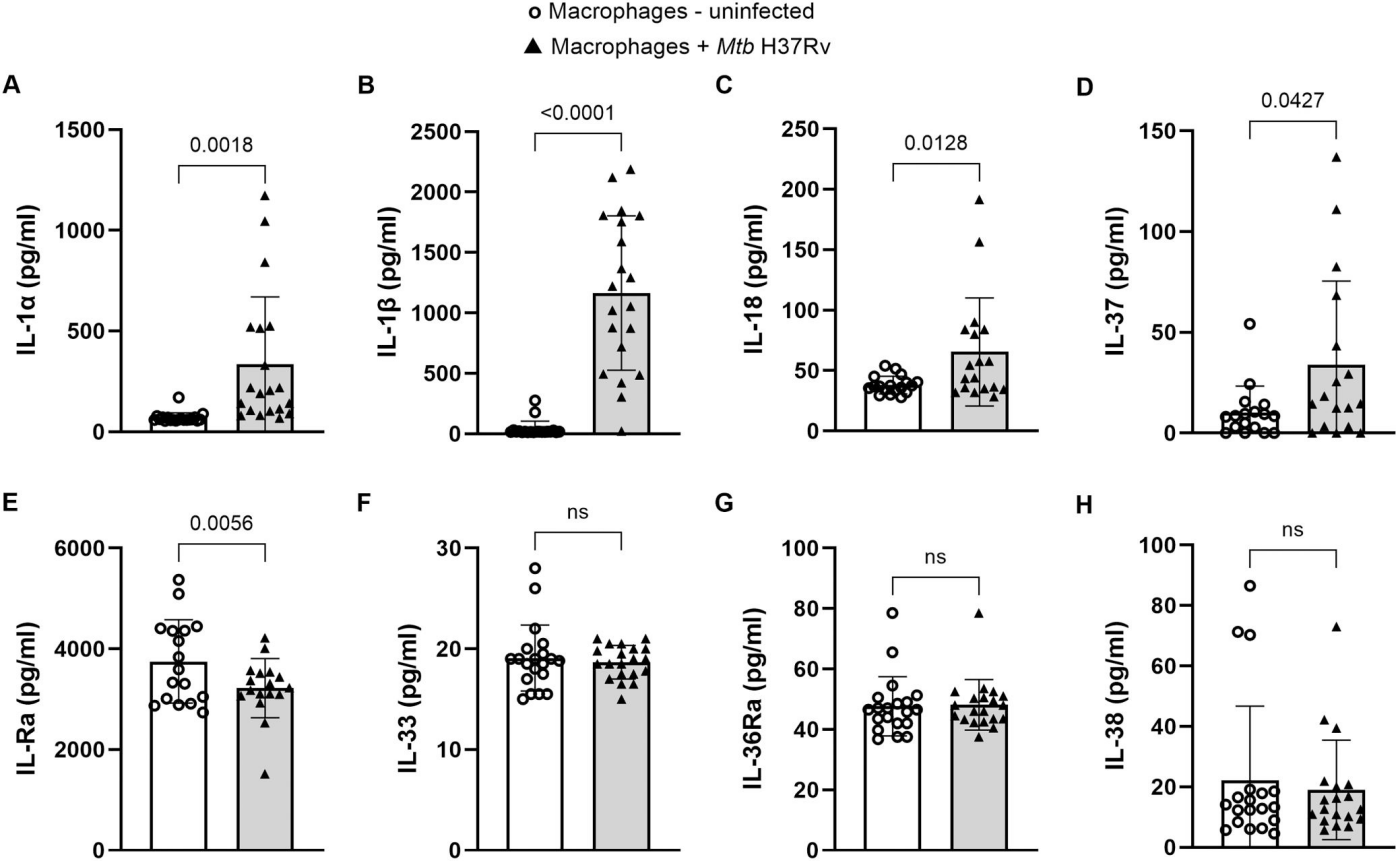
ILSF cytokine production by *Mtb* H37Rv–infected MDMs. Human CD14^+^ monocytes were isolated from 17 to 20 healthy donors, converted to MDMs, and infected with *Mtb* H37Rv as described in the Materials and methods. After 24 hours, the levels of the cytokines IL-1α (A), IL-1β (B), IL-18 (C), IL-37 (D), IL-1Ra (E), IL-33 (F), IL-36Ra (G), and IL-38 (H) in the culture supernatants were measured via multiplex ELISA. Data are shown as mean ± SD. The statistical analysis was performed via 2-tailed paired *t*-tests, and *P* values are shown. ns, not significant.

Human MDMs were infected with *Mtb* H37Rv as described in the Materials and methods. Cytokine levels in culture supernatants were measured by multiplex ELISA after 24 hours. In 17 to 20 healthy donors, *Mtb* infection significantly increased IL-1α (*P* = 0.0018, Fig. 1A), IL-1β (*P* < 0.0001, Fig. 1B), IL-18 (*P* = 0.0128, Fig. 1C), and IL-37 (*P* = 0.0427, Fig. 1D) production (among the 8 ILSFs measured) by MDMs while reducing IL-1Ra (*P* = 0.0056, Fig. 1E) production compared with that in uninfected MDM culture supernatants. There was no significant difference in IL-33, IL-36Ra, or IL-38 production (Fig. 1F–H) by MDMs after *Mtb* infection.

### Effect of recombinant ILSF cytokines on *Mtb* H37Rv growth in human MDMs

**Figure 2.**
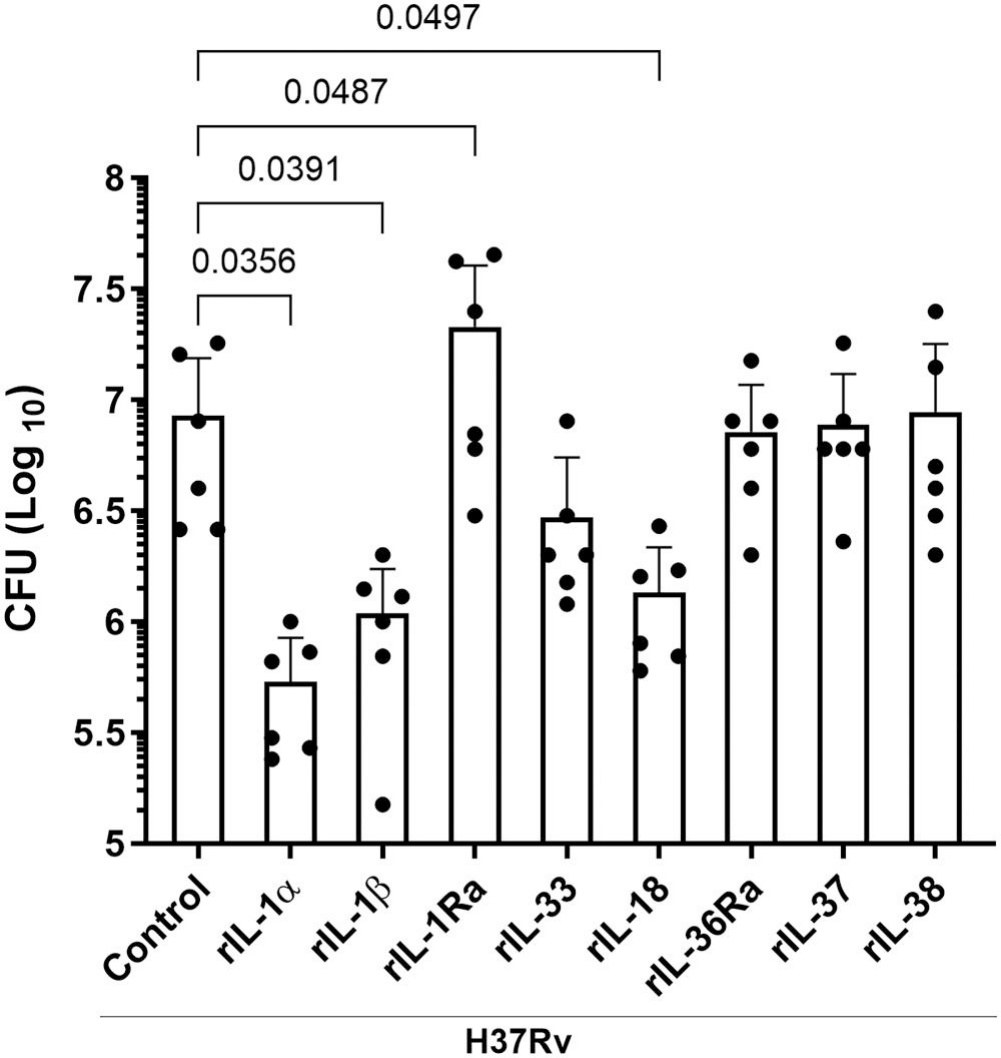
Recombinant ILSF cytokines alter *Mtb* H37Rv growth in human MDMs. Human CD14^+^ monocytes were isolated, converted to MDMs, and infected with *Mtb* H37Rv. Some of the infected MDMs were cultured with various recombinant ILSF cytokines. rIL-1α, rIL-1β, rIL-36Ra, rIL-33, and rIL-38 were used at 10 ng/mL; rIL-1Ra and rIL-18 were used at 100 ng/mL; and rIL-37 was used at 3 µg/mL. After 5 days, the CFUs were determined as described in the Materials and methods. Data are shown as mean ± SD. MDMs from 6 donors were used for this study. The statistical analysis was performed via by repeated-measures one-way ANOVA followed by post hoc Fisher LSD test, and *P* values are shown.

We determined the effects of ILSF cytokines on *Mtb* growth in human MDMs. Some infected MDMs were cultured with or without recombinant ILSF cytokines. After 120 hours, the bacterial burden was measured (Fig. 2). *Mtb*-infected MDMs cultured without any human recombinants from the ILSF were used as controls. Compared with that of the control, the bacterial burden was significantly decreased when *Mtb* H37Rv-infected MDMs were cultured with human recombinant IL-1α (*P* = 0.0356), IL-1β (*P* = 0.0391), or IL-18 (*P* = 0.0497) (Fig. 2). In contrast, compared with the control, the CFUs increased when *Mtb*-infected MDMs were cultured with human recombinant IL-1Ra (*P* = 0.0487). There was no significant difference in bacterial growth between the control and the *Mtb* H37Rv-infected MDMs cultured with rIL-33, rIL-36Ra, rIL-37, or rIL-38 (Fig. 2).

### Neutralization of ILSF cytokine antibodies altered *Mtb* growth in MDMs

**Figure 3.**
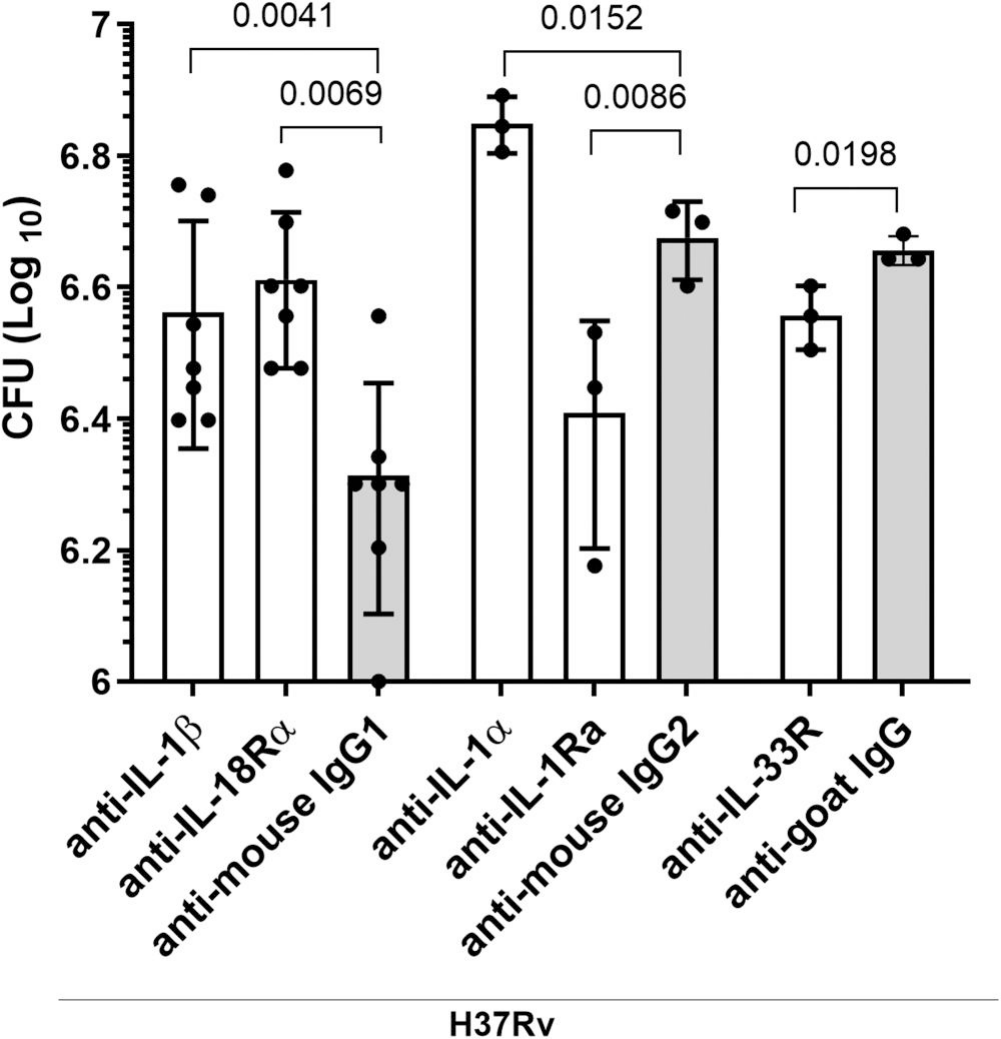
Effect of anti-ILSF cytokines on *Mtb* H37Rv growth in human MDMs. Human CD14^+^ monocytes were isolated, converted to MDMs, and infected with *Mtb* H37Rv. Some of the infected MDMs were cultured with neutralizing antibodies against various ILSF cytokines (10 µg/mL of each cytokine was used). Five days postinfection, the bacterial burden was determined as described in the Materials and methods. Data are shown as mean ± SD. MDMs from 3 to 7 donors were used for this study. The statistical analysis was performed via 2-tailed paired *t*-tests; the respective IgG isotype controls were compared with each neutralization antibody, and *P* values are shown.

The above findings revealed that the IL-1 subfamily cytokines IL-1α, IL-1β, and IL-18 inhibited *Mtb* growth and that IL-1Ra increased the bacterial burden in MDMs (Fig. 2). Next, we determined whether neutralization of these cytokines affects the bacterial burden in *Mtb*-infected MDMs. Some infected MDMs were cultured with and without anti-ILSF cytokine-blocking antibodies. *Mtb*-infected MDMs cultured with IgG isotype control antibodies were used as internal controls. After 120 hours, the bacterial burden was determined (Fig. 3). The bacterial burdens of *Mtb*-infected MDMs cultured with neutralizing antibodies against human IL-1β (*P* = 0.0041; 3.6 × 10^6^ ± 1.3 × 10^6^ vs 2.05 × 10^6^ ± 7.8 × 10^5^), IL-18Rα (*P* = 0.0069; 4.08 × 10^6^ ± 1.09 × 10^6^ vs 2.05 × 10^6^ ± 7.8 × 10^5^), and IL-1α (*P* = 0.0152; 7.06 × 10^6^ ± 7.0 × 10^5^ vs 4.7 × 10^6^ ± 6.4 × 10^5^) were significantly greater than those of their respective control antibodies (Fig. 3). In contrast, bacterial growth significantly decreased when *Mtb*-infected MDMs were cultured with neutralizing antibodies against human IL-1Ra (*P* = 0.0086; 2.56 × 10^6^ ± 9.7 × 10^5^ vs 4.7 × 10^6^ ± 6.4 × 10^5^) and IL-33R (*P* = 0.0198; 3.6 × 10^6^ ± 4 × 10^5^ vs 4.53 × 10^6^ ± 2.3 × 10^5^) compared with the IgG controls (Fig. 3).

### Effect of B cells on ILSF cytokine production by *Mtb* H37Rv–infected monocytes

**Figure 4.**
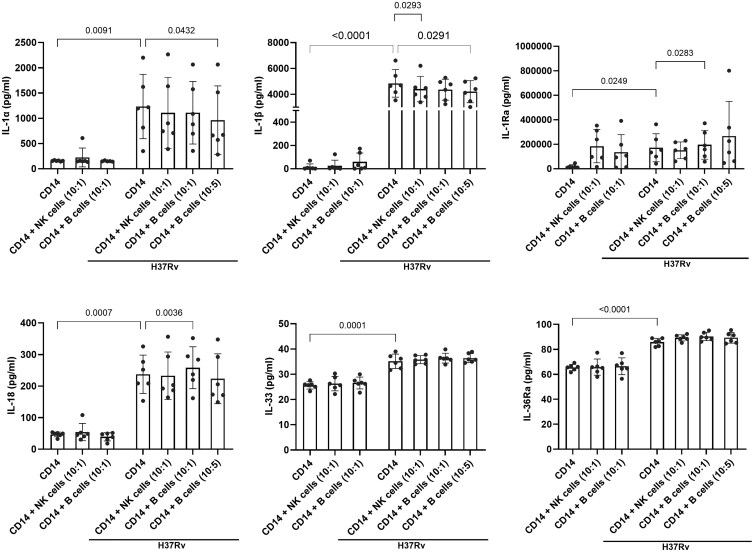
Effect of B cells on ILSF cytokine production by *Mtb* H37Rv–infected CD14^+^ monocytes. Human CD14^+^ monocytes were isolated and infected with *Mtb* H37Rv. Some of the infected monocytes were cultured with different ratios of B cells (monocyte to B-cell ratios of 10:1 and 10:5) or NK cells (monocyte to NK cell ratios of 10:1), as mentioned in the Materials and methods. After 48 hours, the IL-1α, IL-1β, IL-1Ra, IL-18, IL-33, and IL-36Ra levels in the culture supernatants were measured via multiplex ELISA. Data are shown as mean ± SD. Monocytes from 6 donors were used for this study. The statistical analysis was performed via repeated-measures one-way ANOVA followed by post hoc Fisher LSD test, and *P* values are shown.

We determined the effect of B cells on ILSF cytokine production by *Mtb*-infected monocytes. NK cells are known to enhance the functions of *Mtb*-infected monocytes,^29^^,^^38^^,^^39^ so we also cultured *Mtb*-infected monocytes with NK cells as a control. We used freshly isolated CD14^+^ monocytes instead of MDMs and cultured them with autologous B or NK cells to avoid using blood from the same donor twice. Freshly isolated human monocytes were infected with *Mtb* H37Rv, and some of the *Mtb*-infected monocytes were cultured with 2 different ratios of freshly isolated B cells (10 monocytes with one or 5 B cells) (Fig. 4 and Fig. S1A, B). Some of the *Mtb*-infected monocytes were cultured with freshly isolated NK cells (10 monocytes with one NK cell) (Fig. 4). After 48 hours, the levels of the cytokines IL-1α, IL-1β, IL-1Ra, IL-18, IL-33, IL-36Ra, IL-6, IL-10, and TNF-α in the culture supernatants were measured via ELISA (Fig. 4 and Fig. S1C). Compared with uninfected monocytes, after 48 hours, *Mtb*-infected monocytes without B cells produced significantly greater amounts of IL-1α (*P* = 0.0091), IL-1β (*P* < 0.0001), IL-1Ra (*P* = 0.0249), IL-18 (*P* = 0.0007), IL-33 (*P* = 0.0001), IL-36Ra (*P* < 0.0001), IL-6 (*P* = 0.0130), IL-10 (*P* < 0.0001), and TNF-α (*P* = 0.0131) (Fig. 4 and Fig. S1C). Although *Mtb* infection reduced IL-1Ra production by MDMs after 24 hours (Fig. 1E), IL-1Ra production increased after 48 hours (*P* = 0.0249, Fig. 4) compared with that in uninfected monocytes. Coculturing B cells (10 monocytes with 5 B cells) with *Mtb*-infected monocytes for 48 hours resulted in reduced production of IL-1α (*P* = 0.0432), IL-1β (*P* = 0.0291), IL-6 (*P* = 0.0197), and TNF-α (*P* = 0.0181) compared with that in *Mtb*-infected monocytes without B cells (Fig. 4 and Fig. S1C). In contrast, coculturing B cells (10 monocytes with one B cell) with *Mtb*-infected monocytes for 48 hours resulted in increased production of IL-1Ra (*P* = 0.0283) and IL-18 (*P* = 0.0036) compared with that of *Mtb*-infected monocytes without B cells (Fig. 4). A similar increase in IL-10 production (*P* = 0.0310) was noted when 10 monocytes and 5 B cells were cocultured with *Mtb*-infected monocytes for 48 hours (Fig. S1C). There was no significant difference in IL-33 or IL-36Ra production between *Mtb*-infected monocytes and *Mtb*-infected monocytes cultured with B cells (Fig. 4). NK cells reduced IL-1β (*P* = 0.0293) and IL-10 (*P* = 0.0278) production by *Mtb*-infected monocytes (Fig. 4 and Fig. S1C). NK cells had no effect on the production of other ILSF cytokines, and IL-6 and TNF-α, by *Mtb*-infected monocytes (Fig. 4 and Fig. S1C).

### Effect of B cells on *Mtb* H37Rv growth in human monocytes

**Figure 5.**
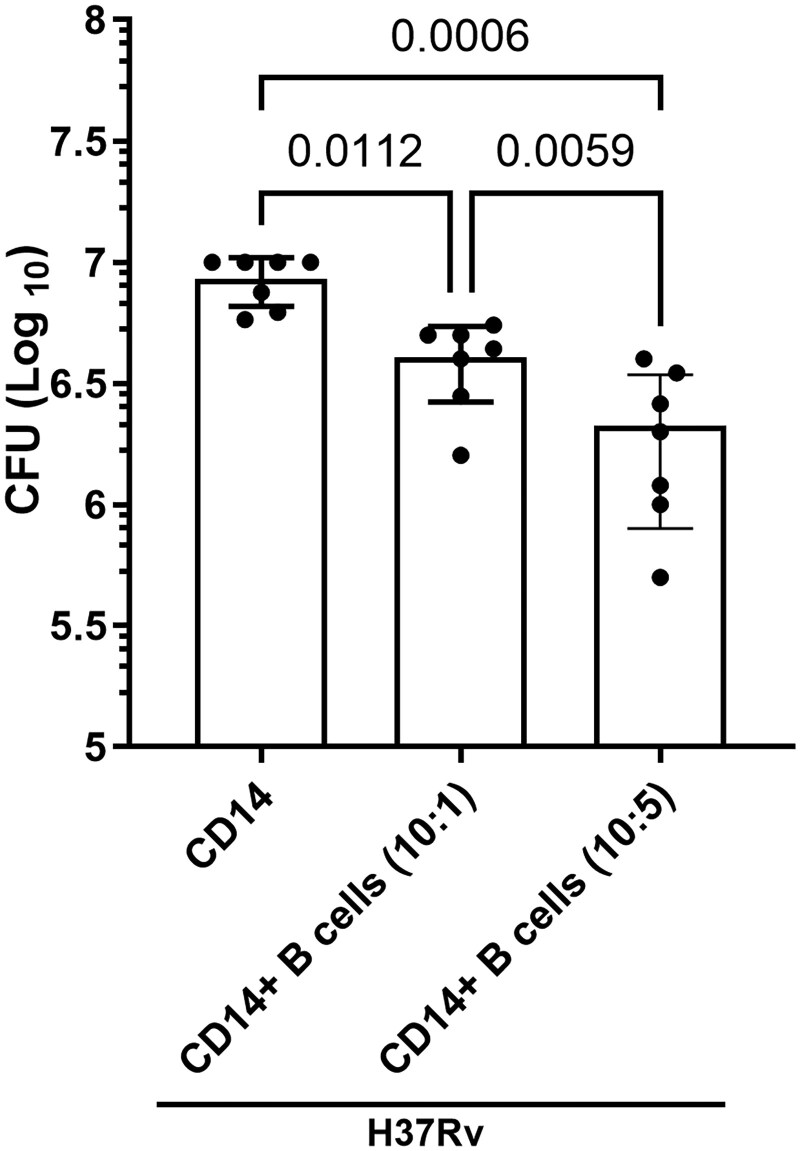
B cells reduce the bacterial burden of *Mtb* H37Rv–infected monocytes. Human CD14^+^ monocytes were isolated and infected with *Mtb* H37Rv, and some of the infected monocytes were cultured with different ratios (monocyte to B-cell ratios of 10:1 and 10:5) of B cells, as mentioned in the Materials and methods. After 120 hours, the bacterial burden was determined. Data are shown as mean ± SD. Monocytes from 7 donors were used for this study. The statistical analysis was performed via repeated-measures one-way ANOVA followed by Tukey multiple comparisons test, and *P* values are shown.

We determined the effect of B cells on *Mtb* growth in monocytes. In this experiment, some of the *Mtb*-infected monocytes were cultured with 2 different ratios of freshly isolated B cells (10 *Mtb*-infected monocytes with one or 5 B cells) (Fig. 5). As a control, some *Mtb*-infected monocytes were cultured for 120 hours without B cells. We found that B cells significantly reduced *Mtb* H37Rv growth in monocytes compared with that in monocytes infected with *Mtb* alone (Fig. 5). Culturing *Mtb*-infected monocytes with more B cells reduced *Mtb* growth more efficiently. (10:1 ratio, *P* = 0.0112; 10:5 ratio, *P* = 0.0006) (Fig. 5).

### B cells increased death pathway gene expression in *Mtb* H37Rv–infected human monocytes

**Figure 6.**
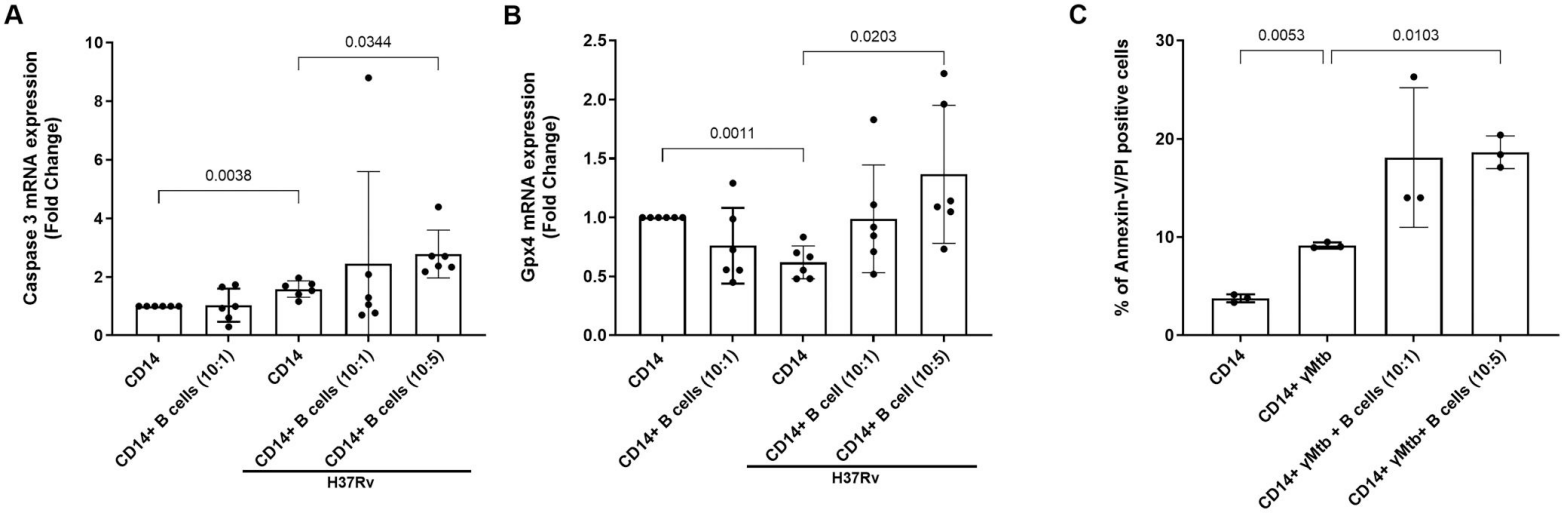
*Mtb* stimulation alters cell death pathway gene expression and apoptosis of human monocytes in the presence of B cells. Human CD14^+^ monocytes were isolated and infected with *Mtb* H37Rv. Some of the infected monocytes were cultured with B cells at different concentrations (monocyte to B-cell ratios of 10:1 and 10:5). (A and B) After 48 hours, the expression of genes involved in various death pathways was determined via qRT-PCR. Monocytes and B cells from 6 donors were used for this study. (C) Monocytes were cultured with B cells (monocyte to B-cell ratios of 10:1 and 10:5) and stimulated with γ-*Mtb* at 10 µg/mL. At 48 hours poststimulation, apoptosis was determined via Annexin V/PI staining. Data are shown as mean ± SD (n = 3). The statistical analysis was performed via repeated-measures one-way ANOVA followed by post hoc Fisher LSD test, and *P* values are shown.

We determined whether B cells enhance the apoptosis of *Mtb*-infected monocytes to restrict their growth. Uninfected and *Mtb*-infected monocytes were cultured with or without B cells. B cells were added at 2 different concentrations (10 *Mtb*-infected monocytes with one or 5 B cells) (Fig. 6). After 48 hours of *Mtb* infection, expression of various genes involved in cell death was determined via qRT-PCR. As shown in Fig. 6A and B, culturing *Mtb*-infected monocytes with B cells (10 *Mtb-*infected monocytes with 5 B cells) significantly upregulated Caspase 3 expression (*P* = 0.0344) and GPX4 expression (*P* = 0.0203). There was no difference in the expression of LC3B, Caspase 1, RIPK3, or MLKL among the groups (Fig. S2A–D). Furthermore, we found that B cells enhance (10 *Mtb-*infected monocytes with 5 B cells) the apoptosis of γ-*Mtb*-cultured monocytes, as determined by Annexin V/PI staining (*P* = 0.0103; Fig. 6C and Fig. S2E).

## Discussion

ILSF cytokines play important roles during *Mtb* infection.^4^^,^^7^^,^^40^^,^^41^ However, only limited information is available concerning the comprehensive role of these cytokines during human *Mtb* infection.^7^^,^^42^ In the present study, we found that compared with uninfected MDMs, human MDMs infected with *Mtb* produced significantly higher levels of IL-1α, IL-1β, IL-18, and IL-37 and lower levels of IL-1Ra. Human recombinant IL-1α, IL-1β, and IL-18 significantly reduced *Mtb* growth in MDMs while human recombinant IL-1Ra enhanced its growth. Furthermore, we found that neutralization of the cytokines IL-1α, IL-1β, and IL-18α significantly increased the bacterial burden in *Mtb*-infected MDMs. In contrast, neutralization of IL-1Ra and IL-33R significantly reduced the bacterial burden. We also found that B cells significantly increased IL-1Ra, IL-18, and IL-10 production and reduced IL-1α, IL-1β, IL-6, and TNF-α production and the bacterial burden in *Mtb*-infected monocytes.

ILSF cytokines play distinct roles in various diseases.^10^^,^^43^^,^^44^ It is well known that innate immune cells produce the IL-1 family of cytokines and exhibit protective immunity against TB.^7^^,^^20^^,^^45–47^ IL-1α/β double knockout mice infected with *Mtb* exhibited larger granulomatous lesions and greater bacterial loads in their lungs.^48^ Similarly, IL-1R^−/−^ mice are highly susceptible to *Mtb* infection through a process mediated by IL-1α production.^12^ In the present study, we found that MDMs infected with *Mtb* secreted significantly higher levels of IL-1α, IL-1β, IL-18, and IL-37 and lower amounts of IL-1Ra than uninfected MDMs did (Fig. 1). Control and *Mtb*-infected MDMs produced similar amounts of IL-33, IL-36Ra, and IL-38. IL-1α, IL-1β, IL-18, and IL-37 production depends on caspase or inflammasome activation.^15^^,^^49–52^ Our findings suggest that early *Mtb* infection (24 hours after infection) initiates caspase- or inflammasome-dependent events that initiate a proinflammatory reaction to eradicate *Mtb* in macrophages.

Limited information is available about the effects of recombinant and anti-IL-1 cytokines on various diseases and infections, including *Mtb* infection.^53^ In *Mtb*-infected MDMs, human recombinant IL-1α, IL-1β, and IL-18 reduced *Mtb* growth while human recombinant IL-1Ra enhanced *Mtb* growth (Fig. 2). Blocking antibodies against IL-1α, IL-1β, or IL-18 enhanced *Mtb* growth in MDMs. In contrast, blocking antibodies against IL-1Ra or IL-33R decreased *Mtb* growth in MDMs (Fig. 3). Exogenous addition of IL-1α and IL-1β was reported to reduce mycobacterial growth in macrophages.^54^ Using animal models, several other studies have demonstrated the essential roles of IL-1α, IL-1β, and IL-18 in the control of *Mtb* infection.^54–57^ IL-18 plays a protective role in both mice and humans.^17^^,^^18^^,^^58^ Taken together, our findings demonstrate that IL-1α, IL-1β, IL-18, and IL-1Ra have similar effects in both animal models and human MDMs.

Although IL-1Ra, IL-1α, and IL-1β are found in the cytoplasm, the anti-inflammatory effects of IL-1Ra differ from those of IL-1α and IL-1β.^16^ Excess amounts of IL-1Ra increase the susceptibility of hosts to *Mtb* infection.^14^^,^^15^ Our current findings further demonstrate that IL-1Ra can enhance *Mtb* growth in human MDMs (Fig. 2). Previous studies have demonstrated that IL-33 can clear ongoing *Mtb* infection.^59^ Recombinant IL-33 moderately reduced *Mtb* growth in MDMs (Fig. 2), and anti-IL33R also reduced *Mtb* growth in MDMs (Fig. 3), suggesting that excess IL-33 can function as a proinflammatory cytokine. Murine macrophages produce IL-36 upon *Mtb* infection, and IL-1β and IL-18 can also induce the production of IL-36; IL-36 signaling then enhances antimicrobial peptide production and *Mtb* growth inhibition.^60^ IL-36 also enhances antimicrobial peptide production through cholesterol metabolism in the THP-1 cell line.^61^ However, we found that the inability of human MDMs to produce IL-36Ra and treatment with exogeneous recombinant IL-36Ra had no effect on *Mtb* growth, demonstrating the differences among mouse, THP-1 and human studies.^60^^,^^61^

IL-37 is highly expressed in various autoimmune diseases, such as rheumatoid arthritis, systemic lupus erythematosus, and Sjögren syndrome.^40^ Compared with those in healthy donors, plasma IL-37 levels are higher in TB patients, and IL-37 inhibits the phagocytic capacity of *Mtb*-infected THP-1 cells and induces the M2 phenotype.^62^ We found that *Mtb*-infected human MDMs produce significant amounts of IL-37 and that human recombinant IL-37 has no effect on *Mtb* growth in human MDMs (Fig.1 and Fig. 2). IL-38 plays a role in viral infections such as hepatitis B and C,^63^ and the plasma IL-38 levels of type 2 diabetes patients with latent TB infection are significantly higher than those of individuals without latent TB infection. In the present study, *Mtb*-infected and uninfected human MDMs produced equal amounts of IL-38, and recombinant IL-38 had no effect on *Mtb* growth in MDMs (Figs. 1 and 2).

B cells play multiple roles depending on the circumstances. For example, IL-1Ra deficiency can influence the actions of a subtype of B cells on the immune system’s proinflammatory cascades.^64^ B cells can mature through the help of IL-1α and IL-1β stimulation.^65^ Regulatory B cells (Bregs) secrete IL-10 to suppress the human immune system, resulting in the control of some autoimmune diseases.^66^ These findings highlight the dual status of B cells, which function as chaperones of both the pro- and anti-inflammatory responses. In this current study, compared with *Mtb*-infected monocytes without B cells, the culture of B cells with *Mtb*-infected monocytes significantly reduced the production of IL-1α, IL-1β, IL-6, and TNF-α; increased the production of IL-1Ra, IL-18, and IL-10 (Fig. 4 and Fig. S1C); and reduced *Mtb* growth (Fig. 5). For the B-cell coculture experiments, we used monocytes instead of MDMs for autologous culture to avoid using blood from the same donor twice. Recent studies have demonstrated that B cells play multiple roles in *Mtb* infection.^67–72^ During chronic *Mtb* infection, a significant number of B cells are found on the boundaries of granulomas.^5^ B cells have dual properties during the chronic stages of *Mtb* infection to carry out the chief executive functions of the adaptive immune response. B cells interact with CD4^+^ helper T cells in the granulomas of *Mtb*-infected mice and enhance proinflammatory responses.^21^ The ability of B cells to inhibit these excess inflammatory cytokines is important in *Mtb* granulomas to reduce inflammatory pathology and the spread of infection.^67^ IL-1α and IL-1β can induce B cell maturation, differentiation, and immunoglobulin production, suggesting that B-cell and IL-1 crosstalk occurs.^65^ Our findings further demonstrated that B cells can inhibit IL-1α, IL-1β, IL-6, and TNF-α production and increase IL-1Ra, IL-18, and IL-10 production to suppress inflammation during the chronic stages of human *Mtb* infection (Fig. 4 and Fig. S1C).

In response to *Mtb*, NK cells produce IFN-γ and inhibit *Mtb* growth in monocytes.^31^^,^^39^ Therefore, we also measured ILSF cytokine production by *Mtb*-infected monocytes in the presence of NK cells. NK cells reduced IL-1β and IL-10 production by *Mtb*-infected monocytes but had no effect on the production of other ILSF cytokines, such as IL-6 and TNF-α, by *Mtb*-infected monocytes (Fig. 4 and Fig. S1C). These results demonstrate that NK cells inhibit ILSF cytokine production by *Mtb*-infected monocytes.

B cells are unable to suppress proinflammatory immune responses when there is a deficiency of IL-1Ra.^64^ During the chronic stages of *Mtb* infection, excess IL-1 production can cause pathology and tissue damage.^73^ Antibiotic treatment along with IL-1R blockade can reduce disease severity and inflammation in *Mtb*-infected mice and macaques.^73^ Our findings and the above published studies demonstrated that B cells enhance IL-1Ra production to reduce immunopathology by inhibiting the production of the proinflammatory cytokines IL-1α, IL-1β, IL-6, and TNF-α. Bregs are a unique type of B cell that produce IL-10 to suppress proinflammatory immune responses and are necessary for the proper control of autoimmune disorders.^66^^,^^74^^,^^75^ Proinflammatory cytokines such as IL-1β can activate Bregs.^76^^,^^77^ In the present study, B cells cocultured with *Mtb*-infected monocytes significantly increased IL-1Ra, IL-18, and IL-10 production (Fig. 4 and Fig. S1C), suggesting a possible role of IL-10–producing Bregs in regulating excess inflammation in granulomas.

IL-1Ra is made in 2 forms: a secretory IL-1Ra form and a cytoplasmic IL-1Ra form.^78^ B cells may play a more robust role in activating all forms of IL-1Ra via their interaction with *Mtb*-infected monocytes (Fig. 4). Further studies are needed to determine whether Bregs or B cells initiate a downstream signaling pathway that leads to the activation of IL-1Ra in the cytosol of human monocytes (Figs. 4–6). In the promoter regions of IL-1Ra, secretory IL-1Ra has a TATA box with a similar sequence of nucleotides for NF-kB, but cytoplasmic IL-1Ra does not have a TATA box with that sequence of nucleotides.^79^ The binding of IL-1Ra to its receptor IL-1R1 leads to an anti-inflammatory response.^16^

B cells at 2 different ratios (10:1 and 10:5 [monocytes to B cells]) inhibited *Mtb* growth in monocytes (Fig. 5). Culturing B cells with *Mtb*-infected monocytes significantly increased Caspase 3 and GPX4 mRNA expression and increased apoptosis at a ratio of 10:5 (Fig. 6A–C). We found that B cells cocultured with monocytes at a ratio of 10:5 efficiently inhibited *Mtb* growth compared with those in the 10:1 ratio (Fig. 5). Prior studies demonstrated that a reduced *Mtb* burden was associated with an increased number of B cells and anti-*Mtb* antibodies, such as IgA and IgG.^80^ B cells depleted in nonhuman primate cynomolgus macaques infected with *Mtb* exacerbated the bacterial burden and inflammation.^25^ Mice lacking B cells experienced worsening TB disease due to the recruitment of neutrophils.^81^ During *Mtb* infection, marginal zone B cells (MZB cells) accumulate in the mice lungs and spleen.^82^ These MZB cells provide systemic control of *Mtb* infection and display activated and memory-like phenotypes.^82^

Antibody production is the major function of B cells in various diseases and infections.^83^ Antibody production to enhance monocyte-mediated cytotoxicity and enhance the induction of Caspase 3 expression by *Mtb*-infected monocytes may be the major mechanism by which *Mtb* growth is inhibited (Figs. 5 and 6). It is possible that B cells enhance the Fc portion of IgG1 or IgG3 Abs (constant region) bound to target cells, which may increase FcγRIIA (CD16)–mediated killing of target cells to restrict *Mtb* growth.^84^ However, further studies are needed to understand the B cell–mediated inhibition of *Mtb* growth.

In conclusion, among the 8 ILSF cytokines, 3 (IL-1α, IL-1β, and IL-18) restrict *Mtb* growth in human MDMs, one enhances growth (IL-1Ra), and 4 (IL-33, IL-36Ra, IL-37, and IL-38) play no role in *Mtb* growth. B cells increased IL-1Ra, IL-18, and IL-10 production and inhibited IL-1α, IL-1β, IL-6, and TNF-α production by *Mtb*-infected monocytes. A better understanding of the mechanisms by which ILSF family cytokines affect *Mtb* growth in human alveolar macrophages and the role of B cells in the production of these cytokines may facilitate the development of therapies to prevent or treat TB.

## Supplementary Material

vlaf046_Supplementary_Data

## Data Availability

The data that support the findings of this study are within this article and available from the corresponding author upon reasonable request.
