# Supplementary material for: B cells inhibit IL-1 family cytokine production and Mycobacterium tuberculosis growth in human CD14+ cells
Source: Immunohorizons. 2025 Oct 9;9(11):vlaf046. doi: 10.1093/immhor/vlaf046 (PMC12597884; doi:10.1093/immhor/vlaf046)
Supplement: vlaf046_Supplementary_Data [file vlaf046_supplementary_data.pdf]

Supplemental Figure 1 - (corresponding to Fig. 4)

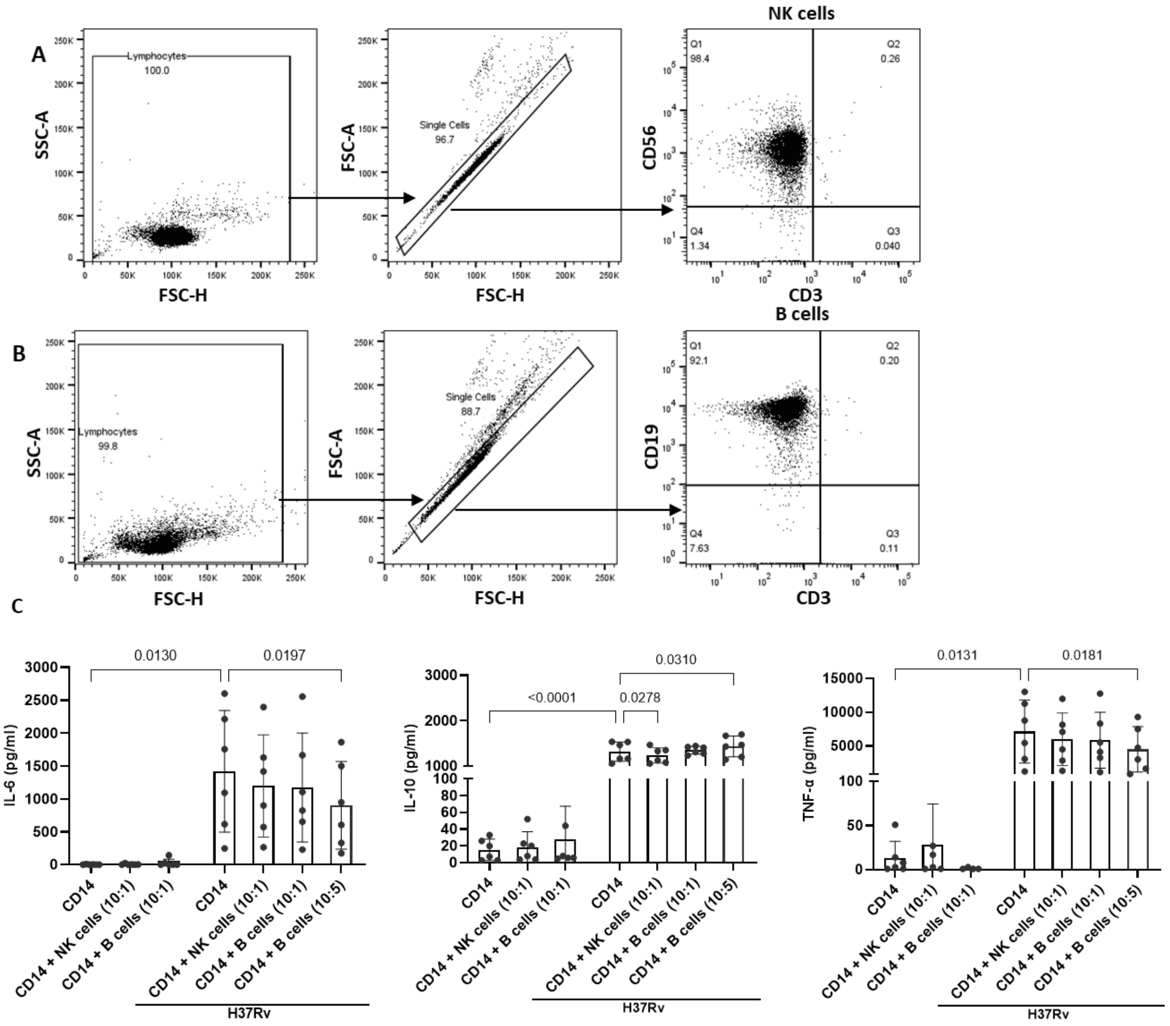

Supplemental Fig. 1. Purity of NK and B cells and effect of B cells on cytokine production by *Mtb* H37Rv-infected monocytes.

(A) Purity of freshly isolated NK cells and **(B)** B cells was determined by flow cytometry, and gating strategies are shown. **(C)** Human $CD14^+$ monocytes were isolated and infected with *Mtb* H37Rv as described in the methods section. Some of the infected monocytes were cultured with different ratios of B cells (monocyte:B cell ratios of 10:1 and 10:5) and NK cells (monocyte:NK cell ratios of 10:1). After 48 h, the IL-6, IL-10 and TNF-α levels in the culture supernatants were measured via multiplex ELISA. The data are shown as the mean ± standard deviations (SD). Monocytes from 6 donors were used for this study. The statistical analysis was performed via repeated measures one-way ANOVA followed by post hoc Fisher's LSD test for multiple comparisons (adjusting for the type I error), and p values are shown.

Supplemental Figure 2 - (corresponding to Fig. 6)

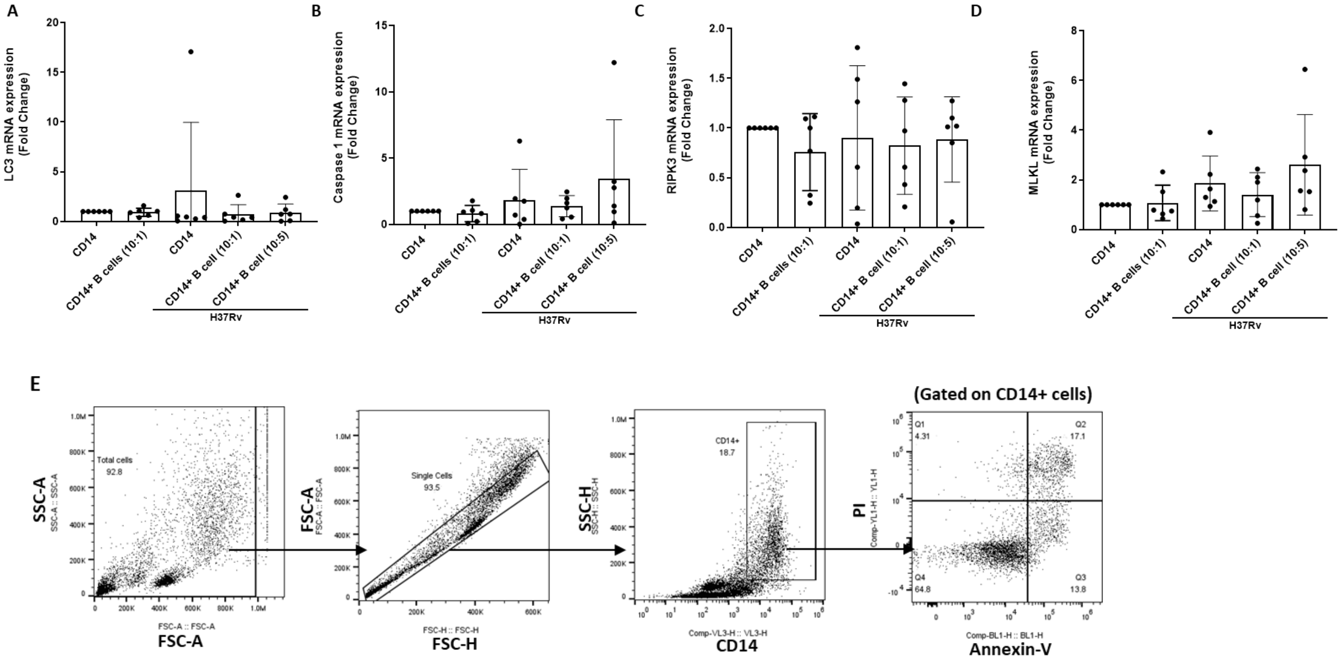

Supplemental Fig. 2. Death pathway-related gene expression and apoptosis measurement in human monocytes in the presence of B cells.

Human CD14^{+} monocytes were isolated and infected with *Mtb* H37Rv, and some of the infected monocytes were cultured with B cells at different concentrations (monocyte:B cell ratios of 10:1 and 10:5). (**A-D**) After 48 h, the expression of genes (LC3, Caspase 1, RIPK3 and MLKL) involved in various death pathways was determined via quantitative real-time PCR. The data are shown as the mean ± standard deviations (SD). Monocytes from 6 donors were used for this study. The statistical analysis was performed via repeated measures one-way ANOVA followed by post hoc Fisher's LSD test for multiple comparisons (adjusting for the type I error). **(E)** After 48 h of γ-*Mtb* stimulation, apoptosis was measured via Annexin-V/PI staining, and representative gating strategy is shown.

Supplemental Table 1. Primers used in this study

Gene	Forward	Reverse
hu Casp3	GGAAGCGAATCAATGGACTCTGG	GCATCGACATCTGTACCAGACC
hu Casp1	GCTGAGGTTGACATCACAGGCA	TGCTGTCAGAGGTCTTGTGCTC
hu Gpx4	ACAAGAACGGCTGCGTGGTGAA	GCCACACACTTGTGGAGCTAGA
hu Lc3b	GAGAAGCAGCTTCCTGTTCTGG	GTGTCCGTTCACCAACAGGAAG
hu Ripk3	GCTACGATGTGGCGGTCAAGAT	TTGGTCCCAGTTCACCTTCTCG
hu Mlkl	TCACACTTGGCAAGCGCATGGT	GTAGCCTTGAGTTACCAGGAAGT
hu β-actin	CACCATTGGCAATGAGCGGTTC	AGGTCTTTGCGGATGTCCACGT
